# Implication of heme oxygenase-1 in the sensitivity of nasopharyngeal carcinomas to radiotherapy

**DOI:** 10.1186/1756-9966-27-13

**Published:** 2008-06-26

**Authors:** Lei Shi, Jun Fang

**Affiliations:** 1 Department of Otorhinolaryngology, the first affiliated hospital of China Medical University, Shenyang, 110001, P. R. China; 2 Laboratory of Microbiology & Oncology, Faculty of Pharmaceutical Sciences, Sojo University, Kumamoto 860-0082, Japan

## Abstract

High expression of the inducible isoform of heme oxygenase (HO-1) is well known in various solid tumors in human and experimental animal models. To investigate the relationship between HO-1 and nasopharyngeal carcinomas, especially its involvement in the response of nasopharyngeal carcinomas to radiotherapy, thirty-two nasopharyngeal carcinomas were semi-quantitatively analyzed by RT-PCR, and the expression of HO-1 was correlated with the consequence after novel radiotherapy, which was evaluated by the reduction of tumor size. Among 32 nasopharyngeal carcinomas, HO-1 expression was found in19 samples (59.4%), in which 9 patients (47.4%) showed no response to radiotherapy. Interestingly, in 13 nasopharyngeal carcinoma patients with negative expression of HO-1, radiotherapy exhibited to be effective (9 patients, 69.2%) or responsive (3 patients, 23.1%). In this study, we first demonstrated the expression of HO-1 in nasopharyngeal carcinomas, and more important, these findings strongly suggest the potential of HO-1as a useful index in identifying patients with well response to radiotherapy, further these data indicate a new therapeutic for nasopharyngeal carcinoma by inhibiting HO-1 activity, which warrants further investigation.

## Background

Heme oxygenase (HO) catalyzes the rate-limiting step of heme degradation, leading to formation of biliverdin, carbon monoxide (CO), and free iron [1,2], which play crucial roles in the adaptation to and/or defense against oxidative stress and cellular stress. HO-1 is a member of the hear shock protein family (HSP-32), and its expression is triggered by diverse stress-inducing stimuli including hypoxia [3], heavy metals [4], UV irradiation [5], reactive oxygen species (ROS) and reactive nitrogen species [5-7]. It is believed that induction of HO-1 protects cells from these toxic stimuli by multiple mechanisms: (a) decreasing the prooxidant level (heme) [8]; (b) increasing the antioxidant level (bilirubin) [9]; (c) producing the antiapoptotic molecule CO [10]; (d) inducing ferritin, which removes and detoxifies free ferric ion [11]; (e) preventing overstimulation of the immune response [12]. Indeed, inhibition of HO-1 by using specific HO inhibitors such as zinc protoporphyrin or tin protoporphyrin extended the pathological consequences of disorders involving these stress-inducing stimuli: graft rejection [13], ischemia-reperfusion injury [14], cisplatin nephrotoxicity [15], and endotoxin-induced septic shock [12]. In contrast, HO-1 inducers such as cobalt protoporphyrin had beneficial effects on certain diseases [14,16].

More important, it is now well known that expression of high levels of HO-1 occurs in various tumors [17], and that HO-1 has an important role in rapid tumor growth because of its antioxidative and antiapoptotic effects [17]. HO-1 was thus considered to be a key molecule for tumors against the attack from the host and chemotherapy and radiotherapy by protecting tumor cells from oxidative insults. It is interesting to note that several tumors, including renal cell carcinoma [18] and prostate tumors [19] in human, express a high level of HO-1. However, very little is known concerning the relationship of HO-1 expression and clinical features in nasopharyngeal carcinomas (NPC). In this study, we have correlated HO-1 expression and the clinical status of nasopharyngeal carcinomas, especially their response to radiotherapy, using RT-PCR analysis.

## Methods

### Patients and tumors

Thirty-two patients diagnosed as NPC from the Department of Otorhinolaryngology at the first affiliated hospital of China Medical University in 2004 and 2005, were selected for this study. Of the 32 patients, the male/female ratio was 20:12 and the mean age was 58.9 years (range 36–78) (Table 1). The tumor specimens were obtained by pharyngoscopical biopsies. No treatments for the malignant tumor were performed prior to this study. Staging of the tumors was carried out according to the TNM classification [20]. The TNM categories were determined by clinical measurement and by a CT scan. The histological grade was examined and classified according to the International Union Against Cancer (UICC) scheme (G1, well differentiated; G2, poorly differentiated; G3, undifferentiated). All patients involved were subjected to radiotherapy (^60^Coγ radiation, 3 Gy each time, one radiation every two days, totally 3 times), one year after which the effect of radiotherapy was evaluated by means of tumor size using clinical measurement and CT scan.

**Table 1:** Correlation of HO-1 expression with clinicopathological features of nasopharyngeal carcinoma


Variable		No. of patients (HO-1 expression)			*P *value ^c^
			
		Total	H + M ^a^	N ^b^	

Age	≤ 61	18	11	7	0.673
	> 61	14	8	6	
Sex	Male	20	13	7	0.873
	Female	12	6	6	
Stage	I + II	13	5	8	0.266
	III + IV	9	14	5	
Histological differentiation	G1	17	7	10	0.174
	G2 + G3	15	12	3	

^a ^Positive (H + M) expression of HO-1.

^b ^Negative (N) expression of HO-1.

^c ^Chi square test, *P *< 0.01 significant.

### Reverse Transcriptase-Polymerase Chain Reaction (RT-PCR) Assay for Expression of HO-1 mRNA in NPC specimens

Total RNA from NPC was extracted by using TRIzol reagent (Life Technologies., Inc., Grand Island, NY), according to the manufacturer's instruction. The RT-PCR assay to detect HO-1 expression was performed according to the method reported by Abraham [21]. The cDNA product obtained by RT with random primers was amplified by PCR. The nucleotide sequences of the oligonucleotide primers used for PCR are as follows: HO-1 antisense 21-mer, 5'GATGTTGAGCAGGAACGCGAT'; HO-1 sense 21-mer, 5' CAGGCAGAGAATGCTGAGTTC' to obtain a 555 bp HO-1 cDNA (nucleotides 79–633 of the coding sequence). After an initial denaturing step at 94°C, 25 PCR cycles were performed as follows: denaturing for 1 min at 94°C, primer annealing for 1 min at 56°C, and DNA synthesis for 1 min at 72°C. The mRNA for glyceraldehyde-3-phosphate dehydrogenase (G3PDH) was examined as a standard mRNA expressed in the cells in the same manner as for HO-1 except that 30 PCR cycles were used. The nucleotide sequences of the primer for RT-PCR for G3PDH are as follows: antisense 24-mer, 5' CATGTGGGCCATGAGGTCCACCAC3'; sense 20-mer, 5' TGAAGGTCGGAGTCAACGGATTTGGT3' to obtain a 983 bp G3PDH cDNA fragment. PCR products then underwent electrophoresis on ethidium bromide-stained 1% agarose gels.

### Semi-quantitative analysis of HO-1 mRNA expression by RT-PCR

**Figure 1:**
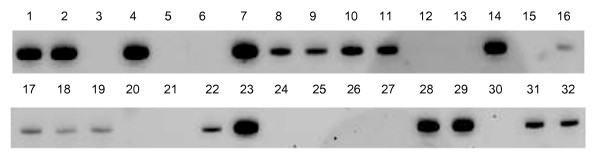
Expression of HO-1 in nasopharyngeal carcinomas. Nasopharyngeal carcinoma specimens were obtained by pharyngoscopical biopsies and were subjected to RT-PCR for determination of HO-1 mRNA expression. HO-1 mRNA expression by RT-PCR was semi-quantified by intensity of DNA band from electrophoresis as negative (N, No. 3, 5, 6, 12, 13, 15, 20, 21, 24, 25, 26, 27, 30), moderate (M, No. 8, 9, 10, 11, 16, 17, 18, 19, 22, 31, 32) and high (H, No. 1, 2, 4, 7, 14, 23, 28, 29) expression.

HO-1 mRNA expression by RT-PCR was semi-quantified by a visual grading system in which the intensity of DNA band from electrophoresis was categorized as negative (N, no or very low expression, no apparent band), moderate (M, apparent band with moderate thickness) and high (H, clear and thick band) expression (Fig. 1).

### Statistical analysis

The statistical significance of differences between HO-1 expression grade and various clinicopathologic features (sex, stage, differentiation etc.) were assessed by the chi-square test for independence. All of the data are expressed as means ±SE. Student's t text was used to determine the significance between each experimental group. The difference was considered statistically significant when *P* < 0.05.

## Results

### Expression of HO-1 in NPC

Among 32 NPCs, 19 samples expressed HO-1 at different degree, as demonstrated by RT-PCR, in which 11 NPCs showed moderate expression of HO-1 and 8 NPCs exhibited high level of HO-1 expression; meanwhile, 13 NPCs (40.6%) showed negative expression (Fig. 1).

### Clinicopathological profiles of the patients related with HO-1 expression

To evaluate the relationship of HO-1 expression with the clinicopathological profiles of NPC, HO-1 expression was classified to two group, positive expression (H + M) and negative expression (N); tumor stage and histological differentiation of tumors were classified as I + II and III + IV, G1 and G2 + G3 respectively. As showed in Table 1, no significant difference of HO-1 expression was found between each investigated group in all observed factors – age, sex, tumor stage and histological differentiation of tumors.

### HO-1 expression associated with the effect of radiotherapy in NPC patients

**Figure 2:**
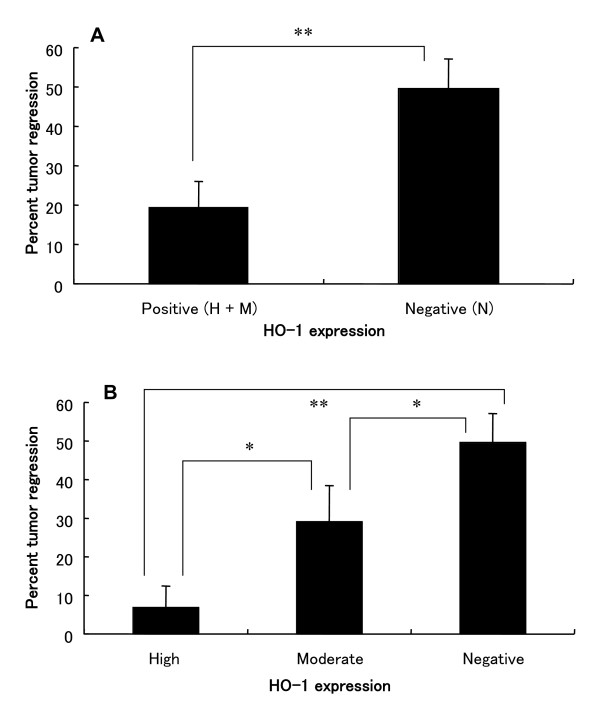
Association of HO-1 expression with the effect of radiotherapy in nasopharyngeal carcinoma patients. A) shows the results between HO-1 negative group (N) and positive group (H + M). The comparison data among each treatment group (H, M and N) is showed in B). Data are described as Mean ± SE. *, *P* < 0.05; **, *P* < 0.0005. See text for details.

Radiotherapy is one of the most important treatments for NPC, by generating cytotoxic reactive oxygen species (ROS) such as singlet oxygen (^1^O_2_), hydroxyl radicals (·OH) etc.. Because of the antioxidative and antiapoptotic role of HO-1, we hypothesized that HO-1 expression may render resistance of NPC patients against radiotherapy, which is a crucial problem in clinic for NPC treatment. To investigate this association, above mentioned 32 NPC patients were subjected to conventional radiotherapy. One year after the treatment, the therapeutic effect was evaluated by the reduction of tumor size as measured by CT scan. As showed in Fig. 2, HO-1 negative patients exhibited an average of 49.6% of tumor regression after radiotherapy, whereas high expression of HO-1 induced a great resistance to radiotherapy, with a significantly decreased tumor regression rate of 6.9% (*P* < 0.0005 vs HO-1 negative group); in between, treatment group with moderate HO-1 expression showed an average tumor regression of 29.1%. These data strongly supported our hypothesis about the association of HO-1 expression with the clinical effect of radiotherapy for NPC.

## Discussion

Nasopharyngeal carcinoma (NPC) is relatively rare in many countries in the world, for example, in U.S. it represents only 0.2% of all malignancies. However, in a number of endemic areas it is the most common tumor found, comprising 18% to 25% of all cancers. These endemic areas include southern China, Hong Kong, Taiwan, Kenya, the Philippines, Singapore, Tunisia, Sudan and Uganda. In contrast to this, the prevalence of NPC in neighboring oriental countries, including Japan is quite low. The incidence of NPC in northern China was very low, however, recently it has been found to be increasing gradually.

The mainstay of treatment of NPC is radiation. This is due both to the inaccessibility of the tumor to surgery, and also to the surprising radiosensitivity of the lesion. NPC is also one of the most challenging tumors for the radiotherapist to treat, because of the close proximity of the tumor to many vital structures. Besides, chemotherapy may be used as an adjunct to radiation therapy, and surgery plays a supportive role in the treatment of NPC. Even though radiotherapy is a relatively effective treatment for NPC in clinic, the 5-year survival rate of NPC patients after radiotherapy is less than 50%, it is thus crucial to study on the factors affecting the treatment, thus finding out a way to increase the therapeutic efficacy for NPC is greatly anticipated.

Regarding to radiotherapy, its anticancer mechanisms are mainly due to ionization of the water molecules to produce highly cytotoxic free radicals (^1^O_2_, ·OH radicals etc.) by photons and the electrons present in the radiation source. Radiation can also affect the processes of the cell cycle necessary for cell growth, cell senescence, and apoptosis. Namely, induction of ROS and apoptosis attributes to the therapeutic effect of radiotherapy. Interestingly, recently it has been reported that HO-1 is a major antioxidative and antiapoptotic molecule [18] and plays a very important role for rapid growth of solid tumor [22,23]. Administration of a specific HO inhibitor zinc protoporphyrin resulted in remarkable increase of intracellular ROS, which subsequently induced the apoptosis to tumor cells [23,24]. Because of the potent antioxidative and cytoprotective role of HO-1, clinical application of the enzyme has also been suggested in various disorders associated with the generation of ROS, including atherosclerosis, hypertension, acute renal injury, toxic nephropathy, transplant rejection, endotoxic shock, chronic obstructive lung diseases, gastrointestinal diseases, Alzheimer's disease, and others [17]. Inhibition of HO-1 by HO inhibitor zinc protoporphyrin or tin portoporphyrin has resulted in the worsening of the disease, whereas administration of HO-1 inducers such as cobalt protoporphyrin, hemin and trinitrobenzene sulfonic acid or HO-1 gene transfer had beneficial effects in certain other diseases [17]. More important, HO-1 expression has been observed in various human cancers [17-19], which suggested the potential association of HO-1 with cancer.

Along this line, in this study, we investigated the expression of HO-1 in NPCs. Similar with other reports regarding HO-1 and cancers [17-19,25], 59.4% NPCs from patients involved in this study were found to express HO-1, in which 25% showed high expression and 34.4% were with low or moderate expression (Fig. 1). The expression of HO-1 in NPCs seems not to relate with tumor stage and histological differentiation (Table 1). According to the above properties of HO-1, we thus hypothesized that HO-1 may play an important role in NPC patients resistant to radiotherapy. As expected, significant decrease of efficacy of radiotherapy was found in NPC patients with high expression of HO-1 (Fig. 2).

## Conclusion

Taken together, these data strongly suggest that expression of HO-1 in NPCs is associated with the sensitivity to radiotherapy, which high expression of HO-1 induces to resistance to radiotherapy. Thus HO-1 may be very useful for the prognosis of NPC patients receiving radiotherapy. More important, it has been reported that chemotherapeutic response of tumor cells by various conventional ROS-generating anticancer drugs and authentic ROS, was greatly enhanced by combination of a water-soluble HO inhibitor polyethylene glycol-conjugated zinc protoporphyrin (PEG-ZnPP) [26,27]. We thus anticipated that, for those NPC patients resistant to radiotherapy, combined treatment by radiotherapy and HO inhibitors, such as PEG-ZnPP, may exhibit superior therapeutic effect, which needs further investigation.

## Authors' contributions

LS carried out the clinicopathological studies, clinical therapeutical studies, and participated in the evaluation of therapeutic effect. JF participated in the design of the study and performed RT-PCR assay and the statistical analysis, and drafted the manuscript.
